# Pash 3.0: A versatile software package for read mapping and integrative analysis of genomic and epigenomic variation using massively parallel DNA sequencing

**DOI:** 10.1186/1471-2105-11-572

**Published:** 2010-11-23

**Authors:** Cristian Coarfa, Fuli Yu, Christopher A Miller, Zuozhou Chen, R Alan Harris, Aleksandar Milosavljevic

**Affiliations:** 1 Department of Molecular and Human Genetics, Baylor College of Medicine, One Baylor Plaza Houston, TX 77030, USA; 2 The Human Genome Sequencing Center, Baylor College of Medicine, One Baylor Plaza, Houston, TX 77030, USA

## Abstract

**Background:**

Massively parallel sequencing readouts of epigenomic assays are enabling integrative genome-wide analyses of genomic and epigenomic variation. Pash 3.0 performs sequence comparison and read mapping and can be employed as a module within diverse configurable analysis pipelines, including ChIP-Seq and methylome mapping by whole-genome bisulfite sequencing.

**Results:**

Pash 3.0 generally matches the accuracy and speed of niche programs for fast mapping of short reads, and exceeds their performance on longer reads generated by a new generation of massively parallel sequencing technologies. By exploiting longer read lengths, Pash 3.0 maps reads onto the large fraction of genomic DNA that contains repetitive elements and polymorphic sites, including indel polymorphisms.

**Conclusions:**

We demonstrate the versatility of Pash 3.0 by analyzing the interaction between CpG methylation, CpG SNPs, and imprinting based on publicly available whole-genome shotgun bisulfite sequencing data. Pash 3.0 makes use of gapped k-mer alignment, a non-seed based comparison method, which is implemented using multi-positional hash tables. This allows Pash 3.0 to run on diverse hardware platforms, including individual computers with standard RAM capacity, multi-core hardware architectures and large clusters.

## Background

The advent of massively parallel sequencing has the potential to dramatically increase our understanding of genomic and epigenomic variation and of their interaction[1]. Serving as markers of paternal and maternal chromosomes in heterozygous loci, single-nucleotide polymorphisms (SNPs) have demonstrated utility to provide information about allele-specific histone marks [2] and to identify differential CpG methylation due to imprinting[3]. Our understanding of the functional consequences of SNPs is largely confined to the less than 1.5% of the genome that codes for amino acid sequences. Increasing our understanding of epigenomically-mediated effects has the potential to elucidate functional consequences of genomic variation within the remaining 98.5% of the genome [4,5]. This requires integrative analyses of genomic and epigenomic variation. Pash 3.0 enables such integrative analyses by achieving the speed required to map in acceptable time the high volumes of reads generated by massively parallel technologies while sensitively detecting DNA-sequence level variation in mapped reads.

Genome-wide epigenomic assays increasingly utilize massively parallel sequencing instead of microarrays[3,6]. One recent example involved whole-genome bisulfite sequencing to reconstruct two human methylomes [7]. The project involved sequencing a total of 4.8 billion reads, or 376 Illumina lanes. A naïve method would be to examine similarity between every basepair. When mapping against the 3 × 10^9 ^nucleotides of the human genome, a total of about 10^21 ^basepair comparisons would be required. The gold-standard Smith-Waterman alignment algorithm[8], which performs such basepair-level comparisons, is therefore not practical even if run on the fastest processors. The still dominant "seed-and-extend" paradigm for fast read mapping emerged during the early Sanger sequencing era and has been implemented in comparison tools such as FASTA[9], BLAST[10], BLAT[11] and SSAHA[12]. These "seed-and-extend" tools perform filtering of potential similarities using k-mer level matches, called "seeds", and limit basepair-level comparisons to the areas around the seeds, thus reducing the total number of basepair-level comparisons while still performing at an acceptable sensitivity level. A comprehensive review of early aligners can be found in [13]. The large increase in the number of sequencing reads brought about by massively parallel sequencing required a further increase in comparison speed. Several new aligners such as MAQ[14], Bowtie[15], BWA[16], and Eland have initially improved the alignment speed by using one or a combination of heuristics, such as limiting comparison to short reads, performing ungapped alignment, or restricting the number of acceptable differences between the reads and reference genome. These heuristics have had a generally negative impact on the ability to map reads onto the large fraction of the human genome that is semi-repetitive and to map reads that carry sequence variants not present in the reference sequence, either due to naturally occurring genomic variants, or due to modifications like bisulfite treatment. Newer versions of such aligners have overcome initial limitations, and are able to map long reads containing both basepair substitutions and indels. For a comprehensive overview of next-generation aligners, we recommend a review by H Li and N Homer [17]

The length of Illumina [18] and 454 [19] sequencing reads has nearly tripled over the past three years, opening opportunities to map more efficiently onto the large fraction of genomic DNA that contains repetitive elements and segmental duplications. These longer read lengths provide sufficient information for mapping onto polymorphic sites and for detection of sequence variation including indel polymorphisms. The mapping of bisulfite-treated reads, which contain less information per basepair due to C-T conversion, also benefits from longer read lengths.

Several specialized tools have been developed for the mapping of bisulfite treated reads, such as mrsFAST[20], BSMAP[21], RMAP-BS[22,23], VerJinxer[24], and BRAT[25]. We compare Pash 3.0 specifically to mrsFAST, BSMAP, and RMAP-BS, and show that Pash 3.0 exhibits speed comparable to that of other bisulfite-seq mapping tools, and has higher sensitivity for mapping of real reads.

To fully exploit the benefit of read lengths exceeding 100 basepairs, it is necessary to account for indel-induced gaps, which requires the ability to perform gapped alignments. Instead of performing Smith-Waterman or other types of alignment by dynamic programming, which is not feasible, Pash 3.0 performs a k-mer level alignment. In other words, instead of performing alignment by comprehensively examining similarity at every basepair, Pash examines multi-basepair similarities, ignoring isolated basepair-level similarities. Specifically, Pash examines similarities involving at least *k *basepairs and, analogous to the dynamic programming alignment algorithm, computes gapped alignments consisting of such similarities. If the score is within a certain range, banded basepair-level dynamic programming alignment is performed to identify the best mapping.

Pash 3.0 implements k-mer level alignment using *multi-positional hash tables*. This data structure generalizes a previous implementation of single-positional hashing [26,27], which limited each hash table to exactly one sampling position. By allowing the hashing of any number of sampling positions, the multi-positional hash tables allow greater adaptability to various hardware configurations, from single CPU machines with limited RAM and disk space to large clusters consisting of multi-core CPUs. Multi-positional hashing is particularly useful for reads-vs-reads and reads-vs-genome mapping, in contrast to the single-positional hashing method employed by Pash 1.0 and 2.0, which is best suited for genome-to-genome comparisons.

## Implementation

### Positional Hashing

The seed-and-extend paradigm features prominently in similarity search. This paradigm originally emerged as a solution for the problem of searching a large database using short queries to detect remote homologies. The key requirements for such applications were speed and sensitivity across large evolutionary distances. Initial seed-and-extend algorithms such as BLAST[10] and FASTA[9] relied on query pre-processing, whereas second-generation algorithms such as BLAT[11] and SSAHA[12] utilized in-memory indexing of genome-sized databases.

In contrast to the seed-and-extend paradigm, positional hashing groups matches between compared sequences. Matches involve multiple collinear basepairs that are obtained by sampling the compared sequences using gapped patterns. Specifically, pattern P is defined by sampled bases {x_1_,...,x_k_}. We say that a match-a gapped k-mer match- is detected between sequences S and T in respective positions i and j if S[i+x_1_] = T[j+x_1_], ..., and S[i+x_k_] = T[j+x_k_]. A seed-and-extend method extends a match by local basepair alignment. In contrast, Pash 1.0[26] and Pash 2.0[27] create a positional hash table for each sampled offset in the reads and group neighboring matches to produce a read mapping score. A Pash user has the option of specifying the gapped pattern. By default, for certain pattern sizes, Pash 2.0 employs the best discriminating patterns obtained via extensive simulation and search in [28]. While older versions of Pash collate the matches, for improved accuracy, Pash 3.0 performs a heuristic alignment of k-mer matches.

### Efficient positional hashing for reads: multi-positional hash table

Previous implementations of Pash used distributed positional hash tables, building one separate hash table per sampling position within read. This approach requires the I/O intensive step of hash table *inversion*, which involves writing to disk sets of reads that map onto a target genomic window. For mapping applications using reads produced by next generation technologies, the storage requirement can approach 90-100 GB, with an overall disk activity of 1-2TB written and read during one Pash 1.0/2.0 execution.

**Figure 1:**
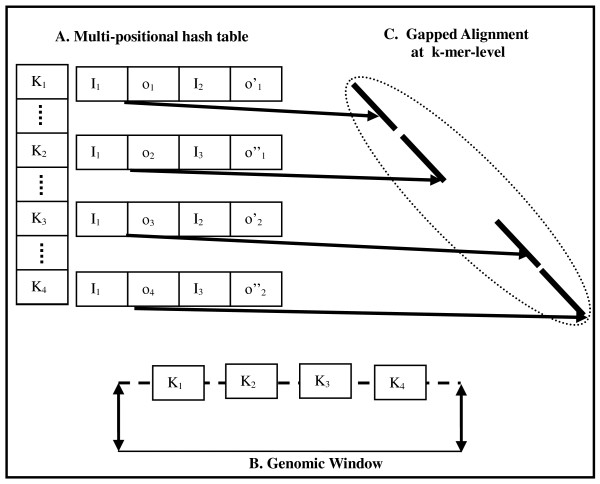
**Multi-positional hash tables**. A. Reads are indexed in a multi-positional hash table. For each k-mer, a multi-positional hash table entry stores the index of the read containing the k-mer, and the offset within the read at which the k-mer occurs. In this example, at k-mers K_1_-K_4 _are present in read I_1_, at offsets o_1_-o_4_, in read I_2 _at offsets o'_1 _and o'_2_, and in read I_3 _at offsets o''_1 _and o''_2_. B. A sliding fixed size genomic window is used; in this example the window contains the k-mers K_1_-K_4_. C. The multi-positional hash table entries for read I_1 _are used to perform k-mer-level alignment.

To avoid this problem, Pash 3.0 employs *multi-positional hash tables*, which in effect combine single-positional hashes at the k-mer level, as presented in Figure 1A. Pash 3.0 generalizes the implementation of positional hashing by removing the constraint that each positional hashing table corresponds to exactly one sampling position (offset). By allowing this multiplicity of offsets, Pash performs better in specific applications (read-genome mapping and read-read mapping) while better adapting to a diversity of hardware resources. When performing read-to-genome mapping, the reads are hashed and Pash streams the target reference against the multi-positional hash tables.

### K-mer level alignment

A crucial step in Pash 1.0 and 2.0 was the collation of neighboring matching k-mers. Pash 1.0 performed ungapped collation of collinear k-mer matches which occur along the same diagonal in the comparison matrix. Pash 2.0 achieved better sensitivity to indels by collating k-mer matches across a small fixed number of neighboring k diagonals using a greedy algorithm. Pash 3.0 goes beyond simple collation and employs a heuristic involving the alignment of matching k-mers in a manner inspired by basepair-level dynamic programming, thus achieving far greater speed, as presented in Figures 1B and 1C. Specifically, Pash 3.0 aligns matching k-mers between a read of size R and a genomic window of size 2W, with 2WR possible seeds as follows:

   Let S = k_1_,k_2_, ...k_p _the matching k-mers between read R

      and genomic window 2W, sorted by offset in the read

   Let t be the number of sampled bases in each k-mer

   Let ovl(d) = number of common positions sampled by two k-basepair patterns that overlap by d bases

   Let m be the reward for a matching base, g the penalty for an indel base

   *Let R = {emptyRun} the set of runs of matching k-mers*

   *bestRun = emptyRun*

   *runExtended = false*

      *foreach i = 1,..,p*

      *k = k*_i_

         *bestExtendScore = 0*

      *foreach run r in R*

         i*f k extends r then*

            *runExtended = true*

            *let d be the overlap length between k and the last k-mer*

               *in run r*

            *let g be the gap size between k and the last k-mer*

               *in run r*

            newScore = score(r)+(t-ovl(d))*m - g * gap_size

            *if (newScore > bestExtendScore) then*

                  *bestExtendScore = newScore*

                  *bestRun = r*

            *end*

            *break*

         *end*

      *end*

      *if (runExtended) then*

         *r = bestRun*

         *append k to r*

         *score(r) = bestExtendScore*

      *end*

   *end*

The running time of the algorithm depends on the density of k-mer sampling. Let G be the offset between k-mer sampling positions. In the worst case, when each k-mer starts a new run, the running time is O((R/G)^2^). Note that for good matches, where most k-mers get added to the same run, the complexity becomes O(R/G). In practice, this k-mer-level alignment is significantly faster than a basepair-level Smith-Waterman alignment.

If the k-mer alignment score for a particular genome window and a read *r *exceeds 3/4 of the current best mapping score for read *r*, then Pash pursues further basepair-level analysis. The first step is to estimate a bounding box for the alignment score. For the best mapping of read *r*, Pash evaluates bounds on an approximate alignment score using an affine function, termed a skeleton alignment score. If the skeleton alignment score exceeds 3/4 of the current best skeleton alignment score for read *r*, a banded dynamic programming alignment is performed. Finally, if the score obtained by dynamic programming is within a user-specified threshold from the best alignment, Pash writes the mapping in a temporary output file. At the end of the alignment, Pash traverses the temporary alignment file and selects the best matches based on the alignment score for each read.

What distinguishes Pash from other algorithms that do nominal seed collation, followed by basepair-level alignment, such as BLAT, is that Pash performs the k-mer-level alignment to enable quick filtering of low quality matches. K-mer-level alignment is a computationally efficient heuristic, since it operates in k-mer space, not at the basepair level.

### Mapping of bisulfite-treated reads

**Figure 2:**
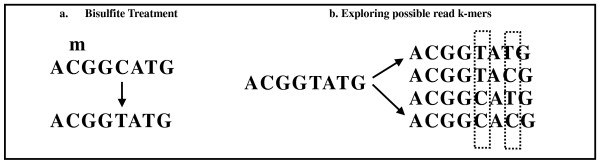
**Mapping bisulfite-treated reads**. A. During the bisulfite treatment, methylated cytosine bases C's are preserved as C's, but unmethylated cytosine bases are converted into uracil and then, upon DNA amplification, into thymine (T's). B. Ts in the reads can correspond to either T's in the reference or unmethylated C's in the reference. For each sequenced k-mer, we explore the space of possible k-mers by converting the Ts into either Cs or Ts. The k-mers are hashed in the read hash table, after which regular mapping is performed.

The k-mer-based framework of Pash enables a straightforward extension to mapping of bisulfite-treated reads. Bisulfite sequencing is an accurate method of determining base-level DNA methylation status [7]. Sample DNA is treated with bisulfite, after which high-volume sequencing is employed. Methylated cytosine bases are preserved, but unmethylated ones are converted to uracil, and typically represented as Ts in the raw sequence, as shown in Figure 2a). There are several challenges in mapping such reads back to a reference genome: 1) Ts can map back to either Ts or Cs in the reference; 2) Reads can come from either the forward or the reverse strand. In Figure 2a we show an example of reads containing both methylated and unmethylated bases, and the effects of applying the bisulfite treatment.

Pash constructs all possible k-mers that may arise from bisulfite treatment by converting the Ts into either Cs or Ts, as shown in Figure 2b. The k-mers are hashed in the read hash table, after which regular mapping occurs. Both the forward and the reverse complement strand of each chromosome are used as a reference for mapping.

### Tradeoffs between speed and sensitivity

Pash can trade speed for sensitivity by modifying the sampling pattern (including k-mer size), k-mer sampling density based on read length, and by ignoring high frequency k-mers. Pash comes with three built-in heuristics: high sensitivity, medium sensitivity, and low sensitivity; for every heuristic the sampling density is adjusted based on read size. Alternatively, a user can specify a specific sampling density. Another means that Pash has for controlling speed is treatment of high-frequency (overrepresented) k-mers. While in Pash 2.0 this was achieved by imposing a hard limit on the number of entries in any positional hash table bin, in Pash 3.0 a user can specify the percent of hashed k-mers to be used for collation. Typically, 99% of the lowest-frequency k-mers are used and the relatively small fraction of 1% high-frequency k-mers are discarded. Pash has another built-in mode, fast, in which the top 7% highest-frequency k-mers are discarded.

### Hardware platform and parameter settings

Our experimental platform consisted of compute nodes with 8-core Intel Xeon X5355 CPUs, 2.66 GHz, and 16 GB of RAM running Linux, kernel 2.6.18. We benchmarked Pash 3.0, BLAT, BWA, BWA-SW, BSMAP, mrsFAST, RMAP-BS, and SSAHA2. All experiments were run sequentially; when the input was split into multiple chunks, we reported total compute time. For Pash 3.0 regular mapping we used the following pattern of weight 13 and span 21: *1110110110001101010*; for bisulfite mapping we used the pattern of weight 12 and span 18 *111010110100110111. *By low-level performance analysis, we determined that Pash 3.0 typically spends only 5-10% of its execution time performing basepair level Smith-Waterman alignment. The most expensive step, consuming roughly 50-60% of its execution time, is the hash inversion step in which all possible k-mer matches between genomic windows and the reads stored in the multi-positional hash table are considered. The memory requirements of Pash were 2-3 GB. Blat used up to 4.2 GB of RAM, BWA and BWA-SW used up to 3.5 GB of RAM, mrsFAST and RMAP-BS used under 1 GB of RAM, SSAHA2 used 6 GB of RAM, and BSMAP used 12 GB of RAM.

## Results and Discussion

### Accurate alignment against the non-unique fraction of the genome

We first evaluated the performance of Pash by mapping reads against the non-unique portion of the genome, which is increasingly accessible due to increasing read lengths produced by massively parallel technologies. Toward this goal, we developed a benchmark that accounts for segmental duplications, an important feature of mammalian genomes, and one particularly prevalent in the genomes of humans and other primates. This benchmark was described in [27]. Briefly, the UD-CSD benchmark models both the *unique *and the *duplicated *fraction of a genome; the duplicated reads go through *coevolution*, gradually acquiring uniqueness by accumulating differences due to independent mutations. Finally, we model the *speciation*; and then the *divergence *(independent accumulation of mutations) of orthologous reads. The divergence can be viewed either as a measure of species divergence in the case of cross-species alignments, or, in the cases of intra-species alignment, as a measure of the local mutation rate. In both cases, any sequencing error would also contribute to the "divergence" rate. The UD-CSD benchmark is parameterized by the number of unique tags k; number of duplicated tags n; the rate of coevolution *x; *and the rate of divergence *y*.

Using this benchmark, we compared the performance of Pash 3.0 to BLAT, a program well suited for fast mapping of longer reads onto the highly repetitive human genome or across close evolutionary distances (such as inter-primate). For our experiment, we chose a read length of 200 bases, started with 90% unique reads and 10% repetitive, and varied the total number of reads from 500,000 to 8,000,000. After the speciation and divergence simulation, we obtained two read sets, r and s, such that each pair of reads r_i _and s_i _have a common 200 bp ancestor; r_i _and s_i _have been evolved from the common ancestor by the process of coevolution, followed by speciation, and then independent divergence. We then employed Pash and BLAT to anchor the read set r onto s, by running each program and then filtering its output such that only best match for each read is kept. Any time a read r_i _is aligned against its counterpart s_i _we count it as true positive; the overall ratio of true positives with respect to the total number of reads considered represents the true positive rate (TPR), and is used as a measure of sensitivity. In addition, we determine the ratio of true positives out of the total numbers of unique mappings reported, termed positive predictive value (PPV), which is a measure of the mapping specificity.

In Figure 3.1 we present the execution times for Pash and BLAT for 25% coevolution and 1% divergence, while in Figure 3.2 we present execution times for Pash and BLAT for 25% coevolution and 5% divergence. Pash was run using a gapped pattern of weight 13 and span 21, and a k-mer offset gap of 12, while for BLAT we used the default settings. In both cases, the agreement between Pash and BLAT, obtained by comparing the numbers of reads mapped to the expected location by each tool, was 99%; Pash and Blat had similar PPV rates within 1%. For up to 1 million reads, execution times for Pash and BLAT are comparable. When the number of reads increases to 2, 4, 8 million reads, however, Pash outperforms BLAT by a factor of 2 to 4.5.

**Figure 3:**
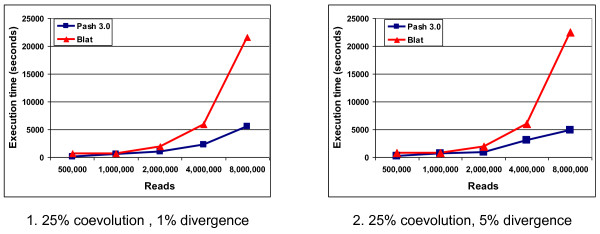
**UD-CSD benchmark results**. 1. Alignment times of Pash and BLAT for coevolution of 25% and divergence of 1%. 2..Alignment times of Pash and BLAT for coevolution of 25% and divergence of 5%.

### Accurate and scaleable alignment of whole genome shotgun sequencing reads

We next compared the performance of Pash to an expanded set of aligners, including those specifically designed for massively parallel sequencing applications. BWA[16] is a recently developed aligner that uses a Burrows-Wheeler transform to index the genome, then performs an index search for matches of reads; for fast execution, a limit on the number of mismatches is set based on the reads size. BWA-SW [29] is a variant of BWA that was tailored for mapping of long reads. SSAHA2[12] is a seed-and-extend aligner, which first builds a "perfect" hash of the genome, and then uses it to seed alignments, and finalizes the alignments using cross_match[30]. For our first experiment, we used simulated human whole genome shotgun (WGS) reads. We obtained them by randomly sampling the human genome (UCSC hg18, NCBI Build 36 http://hgdownload.cse.ucsc.edu/downloads.html) using a uniform distribution. Each basepair was then mutated with a probability of 0.1%, corresponding to the expected human polymorphism rate; 90% of the mutations were basepair substitutions, and 10% were indels in the range 1-10 bp. Next, a uniform sequencing error rate of 2% was simulated. Each read dataset was then mapped onto the reference human genome. To evaluate the performance of mapping, we considered only the reads mapping uniquely, and counted how many of those mapped to a correct location. Table 1 summarizes the execution time, true positive rate (TPR), and positive predictive value (PPV) for Pash 3.0, BWA, BWA-SW, SSAHA2, and BLAT.

**Table 1:** Alignment performance comparison for simulated WGS reads.

Read Size (bp)	76			100			150			200			300		
**Aligner**	**Hrs**	**TPR**	**PPV**	**Hrs**	**TPR**	**PPV**	**Hrs**	**TPR**	**PPV**	**Hrs**	**TPR**	**PPV**	**Hrs**	**TPR**	**PPV**

**BLAT**	15	84.0	97.8	25	86.3	98.7	52.3	87.8	99.4	87.9	88.5	99.6	191.0	89.1	99.7

**BWA/BWA-SW**	0.3	86.7	99	0.8	88.2	99.4	1.9	88.2	99.6	0.9	91	98.3	1.2	91.4	98.7

**Pash 3.0 high**	2.4	87.3	99.1	3.3	88.9	99.4	5.3	89.9	99.5	7.5	90.4	99.5	12.4	90.9	99.7

**Pash 3.0 med**	2	86.8	99.1	2.1	88.3	99.2	2.8	89.7	99.3	3.8	90.4	99.5	6.5	90.8	99.6

**Pash 3.0 low**	1.7	86.7	99.1	1.8	88.2	99.2	2.0	89.6	99.3	2.5	90.3	99.5	4.1	90.8	99.6

**Pash 3.0 fast**	0.7	86.3	98.1	0.8	88.0	98.5	0.9	89.2	98.9	1.1	90.3	99.6	1.0	90.7	99.5

**SSAHA2**	15.3	87.5	99.1	10.6	88.8	99.3	8.8	89.5	99.2	10.0	90	99.2	14.7	90.2	99.0

Execution time, in hours (Hrs), and percent of reads uniquely and correctly mapped to the original location (%U) are reported. Each dataset contains 1 million simulated reads. The read size varies from 76 to 300 bp. Pash 3.0 is run with three different sensitivity settings: high, medium, and low. BWA and BWA-SW are run with default parameters; SSAHA2 is run with the -454 flag, and BLAT is run with default parameters (tile size 11, requiring 2 seeds for a match). We report the true positive rate (TPR), defined as the overall percentage of correctly mapped reads relative to all the input reads, and the positive predictive value (PPV), which indicates what percent of all reported mappings are correct. TPR is a measure of sensitivity, and PPV is a measure of specificity.

Our simulation results show that for reads in the range 76-100 bp BWA is the fastest aligner, faster by a factor of 2.3-8x compared to Pash, but Pash is slightly more sensitive. For 150 bp, BWA and Pash achieve comparable running time, while for reads of length 200 bp and 300 bp BWA-SW has comparable performance to Pash and slightly higher sensitivity. For long reads, Pash is 3.6 to 14.7 times faster than SSAHA2 while achieving comparable sensitivity. Pash outperforms BLAT up to 191x, and achieves higher sensitivity. If time is a constraint, then one can use Pash in fast mode; when accuracy and sensitivity of mapping are at a premium, Pash in high mode should be used.

Next, we used actual Illumina data and 454 reads focusing on comparison between Pash, BWA, SSAHA2, and BLAT. We downloaded a dataset of 19.5 million 76 bp Illumina reads from the 1000 Genomes project website (http://www.1000genomes.org/), run number SRR013932_1, and mapped it with BWA, SSAHA2, and Pash 3.0. For all runs, we determined the reads mapping uniquely. BWA imposes a number of mismatches depending on the read size, and requires the entire read to match. For Pash and SSAHA2, we required reads to match over a length of at least 50 bp, with at most 10% difference between the reads and the reference. The results are summarized in Table 2. BWA is faster by 2.3 to 3.6 times compared to Pash, but Pash maps 23-28% more reads. Note that 96-98% of the Pash mappings agree with the SSAHA2 mappings, whereas only 92% of the BWA mappings agree with the SSAHA2 mappings. For the purpose of mapping near-perfect short reads, or reads that are correctly trimmed to eliminate mismatches due to sequencing error, BWA is an extremely fast and accurate aligner, but relative performance deteriorates with increasing read length, and sequence mismatches due to sequencing error rates or the presence of genomic sequence variants.

**Table 2:** Mapping time and percent of reads uniquely mapped for a 19.5 million 76 bp reads dataset.

Aligner	Execution time (Hrs)	% of reads mapped uniquely
**BWA**	9.2	45.4

**Pash 3.0 high**	33.5	58.2

**Pash 3.0 med**	25.5	56.5

**Pash 3.0 low**	20.5	55.7

**Pash 3.0 fast**	10.7	59.3

**SSAHA2**	234	59.5

Pash 3.0 is run with four different sensitivity settings: high, medium, low, and fast. BWA is run with default parameters. BWA is 1.2-3.6x faster than Pash 3.0, but Pash 3.0 maps 10-14% more of the input reads.

For 454 reads, we compared Pash against BWA-SW, SSAHA2 and BLAT, aligners better suited for long reads. We used the 1000 genomes sample SRR014048, with 1.48 million 454 reads. In Table 3 we present the mapping results, including execution time and percent of reads uniquely mapped with a sequence identity of at least 90%. In high-sensitivity mode, Pash runs 2.6 faster than SSAHA2, and achieves comparable sensitivity, mapping only 0.7% fewer reads. Pash exhibits an effective tradeoff between speed and sensitivity, translating into a graceful sensitivity degradation as the speed increases. At medium sensitivity it maps 1% fewer reads, for a speed increase of 4.7x, and at low sensitivity the number of mapped reads drops by 1.25%, and the execution time decreases 5.95-fold. Pash in fast mode runs 7.9x faster than SSAHA2, at a sensitivity penalty of 0.8% For each sensitivity setting of Pash, 99-99.4% of the Pash mapping agree with the corresponding SSAHA2 mappings. Compared to BLAT, Pash is 15 to 47 times faster; 95.6-95.9% of the reads mapped by Pash are mapped by BLAT at the same location. Finally, Pash achieves a similar speed to BWA-SW on the 454 reads, and maps uniquely 6.3% more of the input reads compared to BWA-SW.

**Table 3:** Mapping performance for a dataset of 1.48 million 454 reads.

Aligner	Execution time (Hrs)	% reads mapped uniquely
**BLAT**	84.5	94.1

**BWA-SW**	1.8	89.8

**Pash high**	5.4	96.2

**Pash med**	3.0	95.9

**Pash low**	2.4	95.7

**Pash 3.0 fast**	1.8	96.1

**SSAHA2**	14.3	96.9

Execution time, in hours, and percent of reads uniquely mapped with at least 90% identity. Pash 3.0 is run with four different sensitivity settings: high, medium, low, and fast. SSAHA2 is run with the -454 flag, and BLAT is run with default parameters (tile size 11, requiring 2 seeds for a match). Pash is 2.6 to 7.9 times faster than SSAHA, with a sensitivity loss of 0.7-1.2%. Pash is 15-47 times faster than BLAT.

### Accurate mapping of bisulfite reads that scales to high-coverage whole-genome bisulfite sequencing

Unlike the mappers discussed above, Pash 3.0 also maps bisulfite-treated reads. To evaluate the performance of Pash 3.0, we compared it with BSMAP [21], the first specialized bisulfite mapping software, mrsFAST[20], and RMAP-BS[22,23]. BSMAP performs bisulfite sequence mapping by hashing the reference genome, and by hashing multiple seeds around the CpG sites, according to the possible methylation status. Our experiments indicate that BSMAP requires a minimum of 12 GB of RAM for the purpose of detecting CpG methylation. We attempted to run BSMAP by hashing multiple seeds around Cs outside of CpG sites as well, but the memory required exceeded 16 GB, the maximum memory on the machines that we used for experiments. Because it does not rely on any assumptions about the location of methylated cytosines, Pash does not have this limitation. Methylation detection outside of CpGs sites is encountered in embryonic stem cells, and can be also used to estimate bisulfite conversion rates. mrsFAST is another aligner capable of mapping bisulfite reads by performing ungapped mapping. In contrast, Pash is sensitive to gaps, which is important for accurate alignments, especially as the read length increases, and is also important for detecting sequence variants that may affect the methylation state or serve as markers to detect allele-specific epigenomic states. RMAP-BS performs gapped mapping of bisulfite-treated reads.

Since BSMAP version 0.99 is limited to mapping reads up to 60 bp, a limitation absent in Pash 3.0, we used two datasets of 60 basepair reads for the purpose of comparison. The first dataset was a simulated WGS dataset over the human genome build hg18: 60 bp reads were randomly picked from the genome, according to a uniform distribution, a 0.1% mutation rate was simulated, with 90% of mutations being a basepair substitution and 10% indels in the 1-10 basepair range. For CpG sites, we simulated methylation with probability of 50%; we did not simulate methylation in non-CpG sites due to the inability of BSMAP to map against them within available RAM. The second dataset was a subset of the whole genome human bisulfite sequencing data set generated by Lister *et al. *[7] trimmed to 60 basepairs. We ran BSMAP using 12 basepairs seeds, and converting reference C's to T's only for C's in CpG contexts. mrsFAST and RMAP-BS were run with default parameters for bisulfite treated reads. Pash was run using gapped seeds of length 18 and weight 12. In Table 4 we show the execution times for Pash, BSMAP, mrsFAST, and RMAP-BS in hours, as well as the true positive rate (TPR) and predictive positive value (PPV), for the simulated dataset. Pash is 2.5-5.2x faster than BSMAP; BSMAP maps 1% more reads. mrsFAST is 1.6-3.4x slower than Pash, and maps 2.7-3% fewer reads than Pash. Pash is 2.3x slower compared to RMAP-BS, and RMAP-BS maps 2.2% more reads. For the trimmed 60 basepairs dataset, Pash is 1.5-3.4x faster than BSMAP, and 1.7-4.1x faster than mrsFAST; and 2.3x slower compared to RMAP-BS. Pash maps 8-11% more actual trimmed reads unambiguously compared to BSMAP, mrsFAST, and RMAP-BS.

**Table 4:** Mapping performance results for simulated and real WGS bisulfite reads by Pash 3.0, BSMAP, mrsFAST, and RMAP-BS.

Aligner	Simulated Reads			Real Reads	
	
	Execution time (hrs)	TPR	PPV	Execution time (hrs)	% reads uniquely mapped
BSMAP	32.1	81.5	99.5	20	72.2

mrsFAST	20.8	77.5	83.9	23.8	72.8

Pash high	12.4	80.5	99.5	13.5	84.4

Pash fast	6.1	80.2	99.1	5.8	81.6

RMAP-BS	2.7	82.7	99.7	2.5	73.2

Each dataset used for evaluation contains 1 million reads. Execution time, in hours is reported for the simulated dataset. We report the true positive rate (TPR), defined as the overall percentage of correctly mapped reads relative to all the input reads, and the positive predictive value (PPV), which indicates what percent of all reported mappings are correct. TPR is a measure of sensitivity, and PPV is a measure of specificity. Execution time, in hours, and percent of reads unambiguously mapped are reported for the real trimmed WGS bisulfite-treated reads. Pash 3.0 is run with two sensitivity settings: high and fast. Pash is 1.5-5.2x faster than BSMAP, 1.6-4.1x faster than mrsFAST, and 2.32x slower than RMAP-BS. On the simulated dataset BSMAP and RMAP-BS are more sensitive, but Pash maps 8-11% more real trimmed reads at unique locations compared to the other aligners.

### Integrative analysis of genomic and epigenomic variation

Epigenomic variation is superimposed on genomic variation, which complicates detection. For example, apparent variation in methylation levels measured in two individuals at a particular basepair position may be either due to the actual variation of methylation levels of a cytosine in that position or, if a SNP occurs in that position, it may be due to variation in genotypes between the two individuals. To further evaluate the performance of Pash 3.0 when simultaneously mapping variation in the genomes and methylomes, we used the data from a recent study on the methylome of H1 and IMR90 [7]. The original publication reports using an in-house bisulfite-seq read mapping method that operates within a 3-letter mapping alphabet, and which relies on the Bowtie aligner [15]. We downloaded the same set of reads and mapped it using Pash 3.0. Only the reads mapping uniquely by either Pash or the 3-letter alphabet method were selected for further analysis. We next removed monoclonal reads and constructed a methylation map including C sites in the genome covered by at least 5 reads. Overall, Pash 3.0 led to methylation calls at an additional 9% of C sites. For the sites where both Pash and the 3-letter alphabet method made calls, the agreement was 99.5%.

Using bisulfite-mapped reads we next identified DNA sequence variation in the H1 and IMR90 genomes. Pash generates read mapping output in the SAM/BAM format [31], which is gaining wide acceptance. Off-the-shelf tools to infer genomic variations from read mappings in the SAM/BAM format, such as SAMtools, are readily available. We ran SAMtools to determine the single nucleotide polymorphism (SNPs) in both the H1 and IMR90 datasets. We discovered 2.4 million SNPs for H1, out of which 88% were found in dbSNP [32], and 2.4 million SNPs for IMR90, out of which 85% were found in dbSNP.

We computed copy number variants (CNVs) using the bisulfite-treated mapped reads. We divided the genome (hg18) into 10 kbp-segments, then counted the number of reads that mapped to each segment of the genome. In order to correct for the Illumina platform's GC content bias, we calculated GC-content for each window, using the methylation map to adjust for the fact that unmethylated Cs are converted to Us by bisulfite treatment. We then binned the GC-content values in increments of 0.01% and applied LOESS correction in R. The data was then segmented into discrete copy-number regions using Circular Binary Segmentation [33]. Windows that were more than 75% covered by repeat regions, or fall into gaps in the reference genome were removed. We used the distribution of reads to set thresholds for homozygous or heterozygous deletion and amplification. Finally, we intersected the resulting putative CNVs with data from 450 normal individuals deposited in the Database of Genomic Variants [34,35]. For H1, 11% of the repeat masked genome is copy number variable, and 64.6% of CNVs overlap with known variants; for IMR90, 10% of the (repeat masked) genome is copy number variable, and 69% of CNV alterations overlap with known variants.

We next performed a two-step integrative analysis of methylation and SNP variation. First, we examined apparent methylation differences between H1 and IMR90. Of all the C sites where H1 and IMR90 differ in apparent methylation levels, 0.4% were SNPs, a four times higher polymorphism rate than the expected 0.1% rate of polymorphism. In the second step, we focused on two groups of SNPs in C positions: those within 19 known imprinted regions and those outside of the imprinted regions, presented in Table 5. We hypothesized that due to the random placement of C/nonC alleles in heterozygotic differentially methylated maternal and paternal imprinted sites in the two cell lines, the ratio of frequency of agreement in methylation levels between H1 and IMR90 in imprinted regions at C/nonC heterozygotic sites vs. C/C homozygotic sites will be significantly less than the same ratio observed in the rest of the genome. As indicated in Table 6, that was in fact the case. The significance of the ratio differences was 0.001 by the chi-square test.

**Table 5:** List of 19 previously known imprinted genes

Gene name
BLCAP
DIRAS3
DLK1
GNAS
GNASAS
GRB10
H19
INPP5F
KCNQ1
MEG3
MEST
NAP1L5
NDN
PLAGL1
PEG3
SGCE
SNRPN
TP73
ZIM2

**Table 6:** Comparison of methylation differences between H1 and IMR90 within 19 imprinted genes and genome-wide.

	C/C vs C/C		C/nonC vs. C/nonC	
	
	Difference in methylation scores	No difference methylation scores	Difference in methylation Scores	No difference in methylation scores
19 imprinted loci	7808	5286	7	4

Genome outside 19 imprinted loci	25170688	16275243	20963	23299

The comparison is performed for C/C sites and for C/nonC heterozygous sites. The ratio of frequency of agreement in methylation levels between H1 and IMR90 in imprinted regions at C/nonC heterozygotic sites vs. C/C homozygotic sites is significantly less than the same ratio observed in the rest of the genome

The methylation maps for H1 and IMR90, the SNPs for both cell lines, as well as copy number variants (CNVs) can be downloaded from http://genboree.org/pash-supp/.

## Conclusions

Pash 3.0 generally matches the accuracy and speed of niche programs for fast mapping of short reads, and matches or exceeds their performance on longer reads generated by new massively parallel sequencing technologies. By using the full sequence information from increasingly long reads produced by massively parallel sequencing technologies, Pash enables analyses of genomic and epigenomic variation within the semi-repetitive fraction of the human genome that will become increasingly accessible as the reads produced by massively parallel sequencing technologies increase in length.

Pash 3.0 uses Positional Hashing, a simple and *transparent *method that gives a user full access to all options that control performance and sensitivity. It can thus be readily reconfigured to accommodate new originally unanticipated applications. It is an *accurate *method that has a low error rate and is both *efficient *and *scaleable*, to match ever-increasing sequencing throughputs. By applying k-mer level alignment, a non-seed comparison method, Pash 3.0 maps accurately to polymorphic sites, including indel polymorphisms. Pash 3.0 also enables epigenomic applications by supporting accurate and fast mapping of bisulfite-treated reads.

We demonstrate the unique capability of Pash to map bisulfite reads for the purpose of simultaneously determining variation in methylation levels and genomic sequence. This capability opens the doors to investigations of allele-specific epigenomic states and also to investigations of the effects of genomic variation on the epigenomic state *in trans *and *in cis*.

## Software availability and requirements

Pash 3.0 runs on Linux and other Unix systems and is available at http://www.brl.bcm.tmc.edu/pash/pashDownload.rhtml. The methylation maps for H1 and IMR90, the SNPs for both cell lines, as well as copy number variants (CNVs) can be downloaded from http://genboree.org/pash-supp/.

## Authors' contributions

CC and AM conceived the Pash 3.0 improvements, designed and interpreted computational experiments, and co-wrote the manuscript. CC implemented Pash 3.0 and performed mapping performance evaluation. FY contributed to the WGS mapping performance analysis. ZC and RAH contributed to the bisulfite-mapping performance analysis. CM performed CNV analysis and evaluation on the bisulfite data. All authors have read and approved the final manuscript.
